# CATalyze-AI: accelerating referral for thrombectomy in acute stroke patients using an AI-based software. A prospective, multicentre, quasi-experimental cohort study

**DOI:** 10.1093/esj/aakag085

**Published:** 2026-07-24

**Authors:** Marta Olive-Gadea, Federica Rizzo, Dolores Cocho, Judit Roca Font, Marc Rodrigo-Gisbert, Marian Muchada, Marta Rubiera del Fueyo, David Rodriguez-Luna, Renato Simonetti, Noelia Rodriguez-Villatoro, Jorge Pagola, Artur Izquierdo, Gemma Planells, Maria Jose Cortes, Maria Àngels Font, Nicoletta Brunelli, Adriano Bonura, Manuel Requena, Jordi Mayol, Ane Murillo, Teresa Jordà-Baleri, Judith Cendrero, Alejandro Tomasello, Carlos Molina, Xabi Urra, Alvaro Garcia-Tornel, Marc Ribo

**Affiliations:** Stroke Unit, Neurology Department, Hospital Universitari Vall d’Hebron, Barcelona, Spain; Stroke Unit, Neurology Department, Hospital Universitari Vall d’Hebron, Barcelona, Spain; Departament de Medicina, Universitat Autònoma de Barcelona, Barcelona, Spain; Neurology Department, Hospital General de Granollers, Granollers, Spain; Emergency Department, Hospital Universitari de Vic, Vic, Spain; Stroke Unit, Neurology Department, Hospital Universitari Vall d’Hebron, Barcelona, Spain; Interventional Neuroradiology, Hospital Universitari Vall d’Hebron, Barcelona, Spain; Stroke Unit, Neurology Department, Hospital Universitari Vall d’Hebron, Barcelona, Spain; Stroke Unit, Neurology Department, Hospital Universitari Vall d’Hebron, Barcelona, Spain; Stroke Unit, Neurology Department, Hospital Universitari Vall d’Hebron, Barcelona, Spain; Stroke Unit, Neurology Department, Hospital Universitari Vall d’Hebron, Barcelona, Spain; Stroke Unit, Neurology Department, Hospital Universitari Vall d’Hebron, Barcelona, Spain; Stroke Unit, Neurology Department, Hospital Universitari Vall d’Hebron, Barcelona, Spain; Neurology Department, Hospital General de Granollers, Granollers, Spain; Emergency Department, Hospital General de Granollers, Granollers, Spain; Radiology Department, Hospital Universitari de Vic, Vic, Spain; Neurology Department, Hospital Universitari de Vic, Vic, Spain; Stroke Unit, Neurology Department, Hospital Universitari Vall d’Hebron, Barcelona, Spain; Stroke Unit, Neurology Department, Hospital Universitari Vall d’Hebron, Barcelona, Spain; Stroke Unit, Neurology Department, Hospital Universitari Vall d’Hebron, Barcelona, Spain; Emergency Department, Hospital Universitari de Vic, Vic, Spain; Stroke Unit, Neurology Department, Hospital Universitari Vall d’Hebron, Barcelona, Spain; Stroke Unit, Neurology Department, Hospital Universitari Vall d’Hebron, Barcelona, Spain; Stroke Unit, Neurology Department, Hospital Universitari Vall d’Hebron, Barcelona, Spain; Grup de Recerca en Ictus, Vall d’Hebron Institut de Recerca, Barcelona, Spain; Emergency Department, Hospital Universitari de Vic, Vic, Spain; Stroke Unit, Neurology Department, Hospital Universitari Vall d’Hebron, Barcelona, Spain; Stroke Unit, Neurology Department, Hospital Clinic, Barcelona, Spain; Stroke Unit, Neurology Department, Hospital Universitari Vall d’Hebron, Barcelona, Spain; Stroke Unit, Neurology Department, Hospital Universitari Vall d’Hebron, Barcelona, Spain

**Keywords:** artificial intelligence, LVO, stroke

## Abstract

**Introduction:**

Timely transfer of patients with suspected LVO remains critical in acute stroke systems. Artificial intelligence (AI)-based imaging tools are increasingly implemented to support triage in non-thrombectomy centres. We assessed whether integrating an AI algorithm within established tele-stroke centres reduces time to transfer decision.

**Patients and methods:**

We conducted a prospective, multicentre, quasi-experimental study comparing consecutive cohorts in 2 tele-stroke centres referring to a single comprehensive stroke centre, before and after implementation of the Methinks Stroke Suite, an AI algorithm for LVO detection on non-contrast CT (NCCT) and CTA. Consecutive transferred patients with suspected acute ischaemic stroke were included. Remote vascular neurologists retained responsibility for final transfer decisions. Each phase spanned approximately 15 months. The primary outcome was time from arrival at the local centre to emergency medical services activation. Secondary outcomes included workflow intervals, imaging utilisation and algorithm performance.

**Results:**

We included 265 patients (136 in the post-implementation cohort; 129 in the pre-implementation cohort). Adjusted median time from arrival to transfer request did not differ (median difference − 2.40 min; 95% CI, −6.16 to 4.48). Post-implementation, time from imaging to transfer request (−7.16 min; 95% CI, −13.02 to −1.85) and arrival to groin puncture (−31.28 min; 95% CI, −60.89 to −13.74) decreased. Computed tomography angiography acquisition at referring centres increased (28%–81%), reducing repeat imaging at the comprehensive centre (79%–42%). Non-contrast CT-based AI prediction yielded a positive predictive value of 66% for endovascular treatment.

**Conclusion:**

Artificial intelligence implementation was not associated with a shorter time to transfer decision. Fewer redundant imaging examinations were associated with a shorter time to reperfusion.

## Graphical Abstract

**Graphical Abstract:**
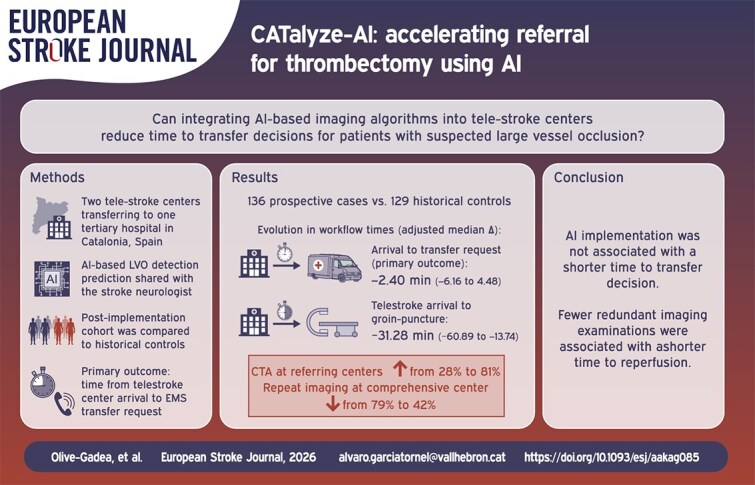

## Introduction

The optimal transfer strategy for patients with a high suspicion of LVO remains debated.^1^ The RACECAT trial, which compared direct transport to a thrombectomy-capable centre vs the drip-and-ship model in Catalonia, yielded neutral results.^2^ However, tele-stroke centres often exhibit slower workflow and treatment times compared to comprehensive stroke centres (CSCs).^3^ A secondary analysis of RACECAT suggested that for patients initially evaluated in centres with poorer time metrics and tele-stroke centres, direct transport to thrombectomy-capable centres may be preferable.^4^

Optimising stroke care by improving time metrics across local stroke centres (local-SCs) could strengthen the entire stroke network, ensure timely reperfusion and improve functional outcomes for all patients. Artificial intelligence (AI) tools have emerged as a promising solution to accelerate imaging interpretation and expedite decision-making. However, stroke networks are highly heterogeneous in terms of workflow organisation and communication infrastructure, and the impact of AI tools is likely to vary depending on these characteristics, requiring their assessment in multiple settings.

The CATalyze-AI study aims to evaluate whether integrating an AI-based image algorithm in the acute evaluation of suspected ischaemic stroke patients assessed in participating local-SC can improve workflow metrics and clinical outcomes, primarily by potentially reducing CTA use and secondarily through facilitating faster image interpretation and triage.

## Patients and methods

This is a prospective, multicentre, quasi-experimental, usual care conditions, open endpoint study of acute stroke patients initially evaluated in local-SC and transferred to a CSC, in which we compared the introduction of the Methinks Stroke Suite in the code stroke workflow of these centres with a pre-implementation cohort.

The study was approved by the ethics committee PR(AG)644/2023 and the study protocol was registered under clinicaltrials.gov (NCT06741332).

A waiver of consent applied to data prospectively collected through mandatory registries.

### Hub-and-spoke description

The study initially planned to include 2 hub-and-spoke models representing both tele-stroke centres referring to a CSC with remote neurologist decision-making, and primary stroke centres referring to a CSC with local management. Due to delays in the primary stroke centre network, the target sample was reached only in tele-stroke centres; thus, this manuscript reports data exclusively from that network.

The participating local-SCs have estimated ground transfer times to the CSC of 30–60 min. One centre serves 385,672 people within an area of 700 km^2^, the other serves 156,972 inhabitants covering 1430.87 km^2^.^5^

Before AI algorithm implementation, suspected acute stroke patients at tele-stroke centres underwent non-contrast CT (NCCT) and remote neurological evaluation by a stroke neurologist after phone-call notification. Non-contrast CT sharing and videoconferencing were conducted via an approved tele-stroke system accessible on computers or a professional smartphone. Non-disabling strokes were typically managed locally and evaluated by a Doppler ultrasound within 24 h. For disabling ischaemic strokes, management depended on clinical severity and imaging availability. Decision on intravenous thrombolysis (IVT) was based on NCCT. Patients were transferred to the CSC in cases of LVO with an indication for endovascular treatment according to current European Stroke Organisation/European Society for Minimally Invasive Neurological Therapy guidelines, or when the consulting neurologist judged CSC transfer to be beneficial. This included situations such as clinico-radiological dissociation, minor stroke with a risk of neurological deterioration (presence of LVO, intra- or extracranial stenosis). Further details on the stroke network structure are provided in the Supplementary methods.

### AI software and implementation of AI protocol

Methinks Stroke Suite (version ce 2.2.1) is a CE-marked radiological computer-aided triage and notification system that uses AI algorithms to identify suspected LVOs on NCCT^6^ and CTA images. The results of the Methinks Stroke Suite are intended to be used in conjunction with other patient information and based on professional judgement to assist with triage/prioritisation of medical images. It reports a sensitivity of 87.2% and specificity of 87.7% for LVO detection on NCCT, and a sensitivity of 92.6% and specificity of 92.9% for LVO detection on CTA.^7^

Before the protocol implementation, the Methinks Stroke Suite was already installed in all participating centres. All urgent cranial CTs were pseudonymised and sent to Methinks’ cloud; predictions were returned in real-time via Microsoft Teams, which also allows for interprofessional communication. Before the protocol start, predictions were not shared between centres.

Beginning in November 2023, the prediction outputs from both participating local-SCs were made available to vascular neurologists from the CSC responsible for the evaluation of suspected stroke cases in the tele-stroke centres. After the implementation of the AI algorithm, the responsible stroke neurologist was encouraged to indicate secondary transfer if the NCCT algorithm indicated a high probability of LVO and the clinician’s judgement deemed the patient as potentially benefiting from EVT without the requirement of additional CTA images. When CTA was performed, its algorithm prediction was also provided to support LVO detection.

### Definition of cohort timeline and eligibility

We compared a pre-implementation and a post-implementation cohort over a period of 15 months excluding a 4-month familiarisation phase. Consequently, the post-implementation cohort began on March 2024, while the pre-implementation cohort included patients evaluated during the 15 months preceding October 2023, with an additional 3-month buffer allowed to balance the number of cases between cohorts.

Post-implementation cohort inclusion criteria included all tele-stroke evaluations performed at the local-SCs for ischaemic stroke adult patients (≥18 years old) within 24 h of symptom onset which were eventually transferred to the CSC as high-priority (stroke code) secondary transfer by emergency medical services (EMS) and whose images were uploaded to the Methinks Stroke Suite platform. Patients presenting beyond 24 h from onset but with fluctuating symptoms were included if a new acute stroke evaluation including neuroimaging was triggered. Patients with central retinal artery occlusion were excluded from the analysis. For in-hospital stroke onset, door time was defined as the time of symptom recognition.

The pre-implementation cohort was obtained from the CICAT mandatory registry,^8^ following the same inclusion criteria as the post-implementation cohort.

### Outcomes

The primary outcome was the change in elapsed time between patient arrival at the local-SC and call to EMS requesting an emergent transfer to the CSC.

Secondary outcomes included additional time metrics (image to EMS transfer request, arrival at the local-SC to groin puncture at the CSC and door-to-needle time in the local-SC), image utilisation (rates of CTA acquisition at the local-SC and rates of repeated imaging at the CSC) and clinical outcomes including mRS score at 90 days and rates of complications during transfer.

Diagnostic accuracy analysis was initially planned. However, because definitive vascular diagnoses were unavailable for a substantial proportion of non-transferred patients, conventional performance metrics were considered potentially biased. To better reflect clinical utility, we report the proportion of transferred patients who ultimately underwent EVT stratified by the algorithm’s predictions. This approach provides a pragmatic measure of how well the tool identifies patients who proceed to thrombectomy in routine practice. Additionally, approximate screening-level estimates were derived from the tele-stroke screening log (Figure S2).

### Statistical analysis

Continuous variables were summarised as medians with IQRs and compared between cohorts using the Mann–Whitney *U* test due to their non-normal distribution. Categorical variables were presented as counts and percentages and compared using the chi-square test or Fisher’s exact test, as appropriate. A 2-sided *P*-value < .05 was considered statistically significant. All variables included in the statistical analyses were complete for the final study cohort; therefore, no imputation procedures were required. For selected baseline characteristics derived from medical records, absence of documentation was treated as absence of the condition.

The association between implementation of the new protocol and the primary outcome—local-SC arrival to EMS notification time—was evaluated using quantile regression estimating the median (τ = 0.5). Models were adjusted for clinically relevant covariates selected a priori based on clinical reasoning and their relevance to stroke workflow, including age, sex, baseline mRS, NIHSS, local-SC, IVT administration, stroke activation time (08:00–17:00 vs 17:00–08:00) and the provider responsible for stroke-code activation (EMS, emergency department or in-hospital). Uncertainty was quantified using rank-inversion 95% CIs, and statistical significance was defined as the absence of 0 within the CI.

A sensitivity analysis using inverse probability–weighted quantile regression estimated the marginal effect while accounting for baseline differences between cohorts. Propensity scores were derived from a logistic regression including age, baseline NIHSS, baseline mRS, IVT, arrival time and the responsible provider for activation, with exact matching on hospital. Nearest-neighbour matching with replacement (1:1 ratio, calliper = 0.2 SD of the logit) was applied. A univariable quantile regression was then performed on the weighted sample to estimate the marginal median effect.

Subgroup analyses were performed for NCCT-based LVO prediction, CTA use, EVT performance and the local-SC where patients were first evaluated. For NCCT prediction, a 3-level exposure variable was created (“pre-implementation” as reference, “post-implementation—high LVO probability,” and “post-implementation—low LVO probability”), and models were fit following the structure of the main analysis. For other subgroups, interaction terms with cohort were included to assess heterogeneity; significance of interactions was evaluated using bootstrap-derived *P*-values.

Secondary time outcomes were analysed using quantile regression adjusted for the same covariates as in the primary model (except for IVT in door-to-needle). Computed tomography angiography utilisation was analysed using multivariable logistic regression to identify patient-level factors associated with acquisition at either centre. Clinical outcomes were assessed using regression models—ordinal logistic for 90-day mRS and binary logistic for transfer-related complications—adjusted for relevant covariates. Odds ratios (ORs) were considered statistically significant if their 95% CIs excluded 1. Algorithm performance was described by reporting the proportion of patients undergoing EVT stratified by NCCT-based and CTA-based predictions.

All analyses were performed using R version 4.3.0 (R Foundation for Statistical Computing, Vienna, Austria).

## Results

**Figure 1:**
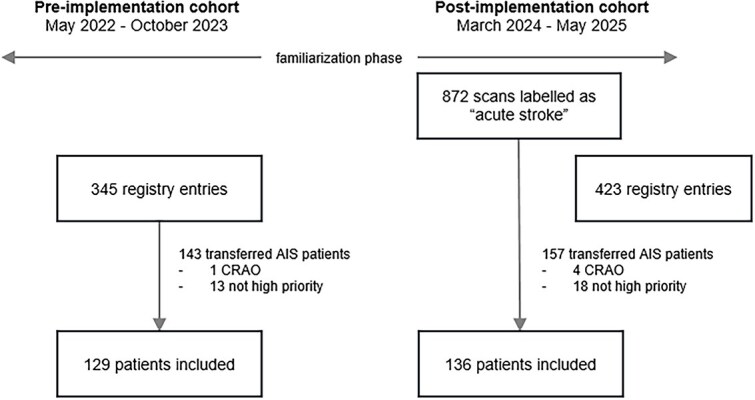
Flowchart of patient identification and inclusion. The pre-implementation cohort was retrospectively identified from a prospective stroke registry, while the post-implementation cohort was prospectively screened at local stroke centres. Only patients transferred as a stroke code by emergency medical services were included. Abbreviations: CRAO = central retinal artery occlusion; local-SCs = local stroke centres.

Between March 2024 and May 2025, 872 neuroimaging scans labelled as suspected stroke were acquired at the 2 participating local-SCs, despite only 423 acute stroke evaluations being prospectively registered in CICAT.^8^ Of these, 157 patients were transferred to the CSC with a clinical diagnosis of ischaemic stroke. Twenty-one patients were excluded (18 due to non-priority transfer and 3 for suspected central retinal artery occlusion requiring ophthalmologic evaluation), leaving 136 patients included in the analysis. Five additional transferred cases were identified but excluded from the analysis because their images were not sent to the Methinks Stroke Suite. Additionally, among 345 acute stroke evaluations recorded in CICAT at the same 2 centres between May 2022 and October 2023, we identified 129 transferred patients who met the inclusion criteria. A flowchart illustrating patient inclusion is presented in Figure 1. Using the number of included patients in the registry as reference, there was no significant difference in the proportion of transferred patients between periods (pre-implementation 37.4% vs post-implementation 37.1%, *P* = .997), or patients receiving thrombectomy (pre-implementation 13.3%, post-implementation 15.4%, *P* = .490). Demographic and clinical characteristics of transferred patients are summarised in Table 1.

**Table 1:** Baseline characteristics.

Variable	Pre (*n* = 129)	Post (*n* = 136)	Total (*n* = 265)	P-value
**Age, median (IQR)**	73 (59–81)	75 (62–83)	74 (61–82)	.165
**Sex (male)**	87 (67%)	81 (60%)	168 (63%)	.183
**Baseline mRS, median (IQR)**	1 (0–2)	1 (0–2)	1 (0–2)	.854
**Hypertension**	55 (43%)	98 (72%)	153 (58%)	<.001
**Diabetes mellitus**	24 (19%)	44 (32%)	68 (26%)	.010
**Dyslipidaemia**	31 (24%)	49 (36%)	80 (30%)	.033
**Coronary heart disease**	9 (7%)	17 (13%)	26 (10%)	.131
**Smoking**	17 (13%)	22 (16%)	39 (15%)	.491
**Atrial fibrillation**	20 (16%)	28 (21%)	48 (18%)	.283
**Prior stroke**	8 (6%)	21 (15%)	29 (11%)	.016
**Local-SC**				
**Centre A**	97 (75%)	88 (65%)	185 (70%)	.063
**Centre B**	32 (25%)	46 (35%)	80 (30%)	
**Responsible of code activation**				.168
**EMS pre-notification**	92 (71%)	82 (60%)	174 (66%)	
**ER, no pre-notification**	28 (22%)	41 (30%)	69 (26%)	
**In-hospital**	9 (7%)	13 (10%)	22 (8%)	
**NIHSS, median (IQR)**	6 (3–13)	6 (3–12)	6 (3–12)	.955
**SBP (mmHg), mean (SD)**	150 (±22)	156 (±27)	154 (±26)	.199
**Glucose (mg/dL), mean (SD)**	136 (±52)	131 (±52)	133 (±52)	.186
**LSW–door (min), median (IQR)**	207 (70–633)	100 (52–280)	131 (56–490)	.003
**Final diagnosis**				.016
**LVO**	40 (31%)	66 (49%)	106 (40%)	
**DMVO**	17 (13%)	14 (10%)	31 (12%)	
**No LVO/DMVO**	61 (47%)	45 (33%)	106 (40%)	
**Mimic**	11(9%)	11(8%)	22 (8%)	
**Intravenous thrombolysis**	40 (31%)	51 (38%)	91 (34%)	.266
**Endovascular treatment**	46 (36%)	65 (48%)	111 (42%)	.045

Abbreviations: DMVO = distal/medium vessel occlusion; EMS = emergency medical services; ER = emergency room; LSW = last time seen well.

Data are presented as *n* (%) unless otherwise specified.

In the post-implementation cohort, NCCT-based predictions were successfully generated for 131 patients (96%), with 62 classified as high-probability LVO (46% of all included cases), whereas 5 cases (4%) resulted in processing errors. Among the 110 patients who underwent CTA, 54 (49%) were classified as high-probability LVO, and output was unavailable in 2 cases (2%) due to processing errors.

### Primary outcome

**Figure 2:**
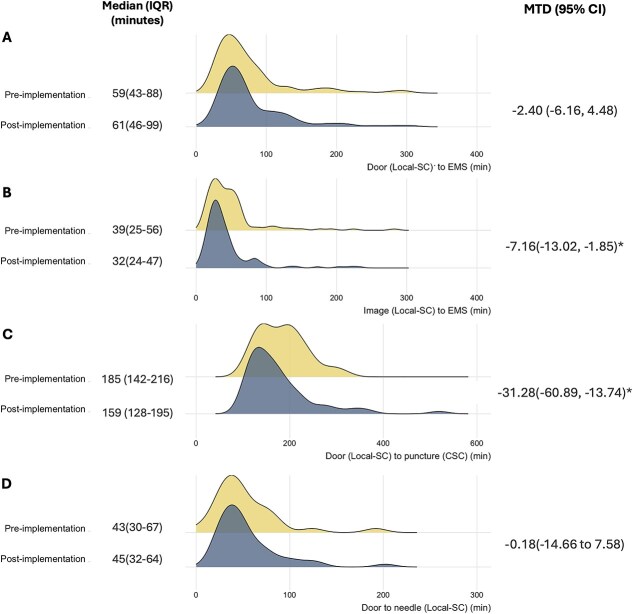
Primary and secondary time-metric outcomes for A) Local-SC arrival to EMS transfer request, B) Image at Local-SC to EMS transfer request, C) Local-SC arrival to groin puncture and D) Local-SC arrival to iv-thrombolysis. ^*^Significant association, defined by 95% CIs excluding 0. Abbreviations: CSC = comprehensive stroke centre; EMS = emergency medical services; local-SC = local stroke centre; MTD = median time difference.

Time from arrival at the local-SC to EMS transfer request did not differ significantly between the pre- and post-implementation cohorts (59 [43–88] vs 61 [46–99] min; adjusted median difference: −2.40; 95% CI, −6.16 to 4.48) (Figure 2A). Coefficients for all model covariates are presented in Table S1.

Balance diagnostics from inverse probability weighting are provided in Table S2 and Figure S1, showing good covariate balance. In the weighted analysis, there was no evidence of an association between cohort and the primary outcome (median difference = −4.00; 95% CI, −23.95 to 5.56).

**Figure 3:**
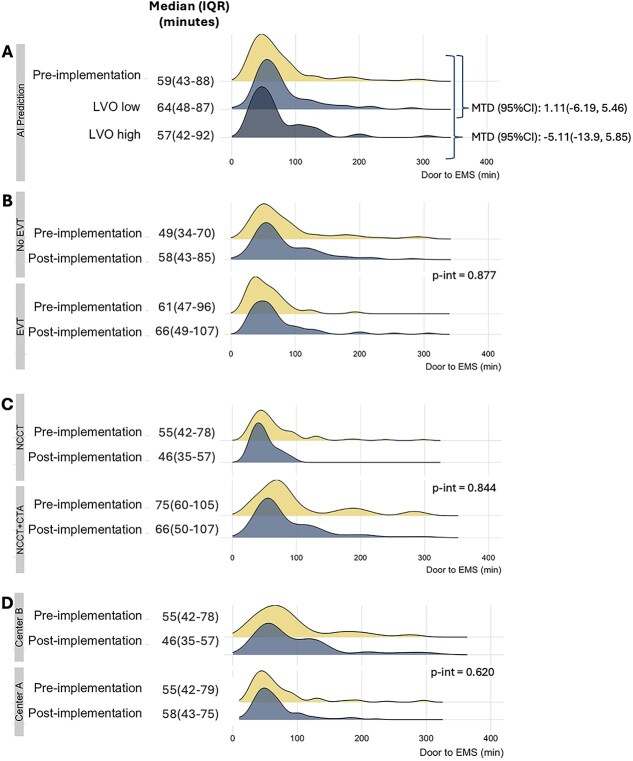
Subgroup analysis for the primary outcome, including A) AI-software prediction (low vs high probability of LVO), B) Endovascular treatment, C) If additonal CTA was performed in the Local-SC and D) Local-SC, Abbreviations: EMS = emergency medical services; MTD = median time difference; NCCT = non-contrast CT.

No significant differences were found in the time from arrival at the local-SC to EMS transfer between the pre-implementation and post-implementation cohorts stratified by NCCT AI high LVO probability (*n* = 62, adjusted median difference = −5.11; 95% CI, −13.9 to 5.85) or low LVO probability (*n* = 70; adjusted median difference = −1.11; 95% CI, −6.19 to 5.46) (Figure 3A). Similarly, no significant heterogeneity was observed according to CTA acquisition (*P*-int = .816), treatment with EVT (*P*-int = .614) or the local-SC where patients were evaluated (*P*-int = .771) (Figure 3B–D).

### Secondary outcomes

In the post-implementation cohort, time from image acquisition and transfer request was shorter (adjusted median difference = −7.16; 95% CI, −13.02 to −1.85), as well as time between arrival at the local-SC and groin puncture (adjusted median difference = −31.28; 95% CI, −60.89 to −13.74). Door-to-needle times at the local-SCs remained unchanged (adjusted median difference = −0.18; 95% CI, −14.66 to 7.58) (Figure 2B–D, Tables S3 and S4).

Computed tomography angiography acquisition at local-SCs increased from 28% (*n* = 33) in the pre-implementation cohort to 81% (*n* = 111) post-implementation (adjusted OR [aOR] = 31.0; 95% CI, 13.30–84.47); the centre of origin was the only other covariate significantly associated with CTA acquisition (Table S5). Consequently, the rate of repeated imaging at the comprehensive stroke centre decreased from 79% (*n* = 102) to 42% (*n* = 57) (aOR = 0.47; 95% CI, 0.22–0.97). Receiving an initial CTA at the local-SC and EVT were both negatively associated with CTA acquisition at the comprehensive centre (Table S6). At 90-day follow-up, the distribution of mRS scores was similar between cohorts (median 2 [IQR 1–4] in both groups; aOR = 1.01; 95% CI, 0.59–1.71) (Table S7). No transfer-related complications were reported in either group.

### EVT rates according to algorithm prediction

Among all patients with a positive NCCT prediction of LVO, 66% underwent EVT and among patients with a positive CTA prediction, 67% underwent EVT (Table 2).

**Table 2:** Association between LVO prediction probability and EVT.

	EVT/High probability predictions	EVT/Low probability predictions
NCCT	66% (41/62)	29% (20/69)
CTA	67% (36/54)	26% (14/54)

Abbreviation: NCCT = non-contrast CT.

Estimates of LVO positive predictive value (PPV) based on screening log data are reported in the Supplementary file (Figure S2; Table S8).

## Discussion

In our hub-and-spoke stroke network, implementing an AI-based triage tool did not shorten transfer decision times. The anticipated benefit of avoiding additional vascular imaging through NCCT-based LVO predictions was not achieved.

Greater CTA use during the study likely reflected a broader availability in local radiology departments. This represents an important confounding factor when interpreting our results, as we cannot isolate the potential impact of AI support on vascular image reading. Subgroup analyses confirmed that time metrics did not differ significantly between pre- and post-implementation cohorts stratified by NCCT prediction categories or local imaging protocols, making it unlikely that the lack of time reduction was due to more vascular imaging studies.

Although there was a reduction in intervals from image acquisition to transfer request and from arrival to groin puncture in the post-implementation cohort, these differences should be interpreted cautiously, as the observed change may reflect a redistribution of tasks. The global reduction in time to groin puncture is likely associated with the increased acquisition of CTA at local centres, which reduced the need for repeated imaging at the CSC.^9^

Small-sample studies suggested that AI algorithms can optimise stroke workflows^10^^,^^11^; however, these findings have not been replicated in larger-scale studies.^12^^,^^13^ Reported benefits may be partially attributed to accompanying communication platforms integrated with these software solutions, rather than the algorithm’s predictive capability alone. In our setting, an immediate image-sharing platform and a well-established tele-stroke circuit were already in place, limiting the potential impact of additional communication tools.^14^ A recent large cohort study from the United Kingdom reported a positive association between an AI stroke suite and higher EVT rates; however, missing workflow and imaging protocol data—and the fact that the control group also had substantial access to AI, with actual use not reported—limit meaningful attribution of these changes to AI.^15^

The study protocol established that the decision to perform additional imaging remained under clinical judgement and did not mandate transfers based solely on NCCT-based AI predictions. Therefore, this study does not evaluate the direct clinical efficacy of the algorithm but rather provides insight into how clinicians interact with AI-generated predictions within a real-world workflow. An interpretation of our results is that clinicians demonstrated a preference for decisions grounded in tangible clinical and imaging findings rather than algorithmic outputs alone. Similarly, the large UK cohort study^15^ reported that in the intervention group AI was used in only 53% of evaluated patients, underscoring a gap with their consistent use in real-world practice. Interaction between clinicians and AI tools is complex and likely influenced by multiple factors, including clinician experience,^16^ the transparency of the algorithm’s decision-making process and the perceived reliability of the tool.^17^ This is especially critical in acute emergency when there is little opportunity for deliberation^18^ as compared with other settings where AI-assisted screening has shown a positive impact.^19^ In our study setting, all patients were evaluated by a stroke neurologist, and clinical expertise was consistently available, which may not reflect how AI tools would be used in settings without access to specialised care.

Commercially available algorithms are expected to generate false positives in one-quarter to one-half of cases when applied to low-prevalence settings (~11%).^20^ Estimating the true prevalence of LVO is challenging due to the absence of a reliable denominator, which is influenced by selection bias in registries.^21^^,^^22^ Our study highlights a striking discrepancy between the number of patients officially recorded and the volume of brain scans submitted for acute stroke evaluation. Although a definitive vascular diagnosis was unavailable for a proportion of non-transferred patients, screening-log analyses allowed estimation of best-case diagnostic performance (PPV) across the entire tele-stroke population (Supplementary file, Table S8). These estimations suggested a modest-to-weak real-world performance and support the concept that AI-based LVO detection tools are most appropriately applied in clinically pre-selected populations with moderate-to-high suspicion of LVO.^20^

These limitations are greatest for NCCT-based predictions, where the clinician only receives a binary result (high vs low probability) without the immediate possibility of verification, whereas CTA-based predictions allow an immediate second look that could lead to the identification of an overlooked occlusion. Given our study design, we cannot exclude a potential benefit of AI-assisted CTA evaluation in settings where CTA is systematically performed.

This study has several limitations, including its non-randomised, quasi-experimental design within a single hub-and-spoke network, limiting causal inference and generalisability; analyses of the protocol’s impact on the likelihood of receiving treatment are limited by the study design, including the different origins of the cohorts. In addition, the small sample size represents another important limitation, potentially reducing the robustness of subgroup analyses. The AI algorithm was implemented as a non-mandatory decision-support tool, reflecting real-world practice rather than the effects of protocolised use; this lack of enforcement can also be viewed as a strength, as it captures how clinicians interact with AI in routine care. Moreover, the study reflects an early phase of algorithm implementation; longer exposure and sustained use may lead to different patterns of clinician adaptation and integration into workflow, potentially yielding different effects over time. Finally, the analysis was limited to transferred patients, and the impact on non-transferred patients could not be assessed.

These methodological limitations highlight key areas for future research on AI-based interventions. First, it is essential to isolate the effect of AI by evaluating the real-world performance of diagnostic-assistant algorithms^23^^,^^24^ and conducting high-quality multicentre studies to distinguish AI effects from contextual factors and assess scalability across diverse settings. Second, clinician-software interaction should be better understood, including how AI influences performance across professionals with different backgrounds and how usability affects real-world adoption. Finally, the safety and cost-effectiveness of AI systems must be critically assessed to establish their true clinical value.

## Conclusion

The implementation of an AI-based triage tool in our stroke network did not reduce transfer decision times. Vascular imaging increased without delaying transfers and was associated with faster treatment among EVT patients. Further research is required to better understand clinician–AI interaction and how these systems can be effectively integrated into clinical decision-making.

## Supplementary Material

ONLINE_SUPPLEMENT_(R3)_aakag085

## Data Availability

The data that support the findings of this study are available from the corresponding author upon reasonable request.
