# Supplementary material for: CATalyze-AI: accelerating referral for thrombectomy in acute stroke patients using an AI-based software. A prospective, multicentre, quasi-experimental cohort study
Source: Eur Stroke J. 2026 Jul 24;11(7):aakag085. doi: 10.1093/esj/aakag085 (PMC13458394; doi:10.1093/esj/aakag085)
Supplement: Supplementary file 1 [file online_supplement_(r3)_aakag085.pdf]

ONLINE SUPPLEMENT

CATalyze-AI: Accelerating Referral for Thrombectomy in Acute Stroke Patients Using an AI-based Software

Table of Content:

Supplemental Methods

- Description of stroke network organizational structure

Supplemental Results

Table S1. Univariate and adjusted multivariate quantile regression for median door to EMS request time

Figure S1. Love-plot summarizing balance of the IPTW-matched cohort

Table S2. Adjusted multivariate quantile regression for median image acquisition to EMS transfer request time

Table S3. Adjusted multivariate quantile regression for median Local-SC arrival to groin puncture at the CSC

Table S4. Adjusted multivariate quantile regression for median door-to-needle time in the Local-SC

Table S5. Adjusted multivariate logistic regression for CTA acquisition at the local stroke center

Table S6. Adjusted multivariate logistic regression for repeated imaging acquisition at the CSC

Table S7. Adjusted multivariate ordinal regression for mRS at 90 days

Figure S2. Detailed LVO predictions from CATalyze cohort and screening log

Table S8. Positive Predictive Value (PPV) of AI LVO predictions

Supplemental Methods

Description of stroke network organizational structure

The region of Catalonia comprises a population of approximately 7.5 million inhabitants across an area of 32,000 km². The Catalan Stroke Network currently includes 6 Comprehensive Stroke Centers (CSC) and 22 Local Stroke Centers (Local-SC), of which 8 are primary stroke centers and 14 are tele-stroke centers.

When Emergency Medical Services (EMS) assess a patient with suspected acute stroke, transfer is typically directed to the nearest stroke center. The standard prehospital severity scale used is RACE. Following publication of the RACECAT trial [2], the regional stroke code protocol was updated to recommend direct transfer to thrombectomy-capable centers for patients at high risk of large vessel occlusion (LVO) (RACE ≥5), in the absence of contraindications to intravenous thrombolysis and when transport time to the thrombectomy center is under 30 minutes.

Of the two participating centers, one is located at a greater distance from the CSC and manages all stroke cases within its catchment area. This center operates exclusively as a tele-stroke center, without an on-site neurology team for emergency care. The second center functions as a primary stroke center during office hours and as a tele-stroke center outside these hours; only tele-stroke cases were included in this cohort. During off-hours, as the neurointerventional team is not physically present at the CSC, the potential time advantage of a mothership model is reduced, and a drip-and-ship strategy remains the standard triage approach.

As described in the main Methods, patients with suspected acute stroke at tele-stroke centers undergo non-contrast CT (NCCT) followed by remote neurological evaluation after phone-call notification. NCCT images and videoconferencing are shared through an approved telestroke platform accessible via computer or professional smartphone (Idonia Health, Palma de Mallorca, Spain). In cases of hemorrhagic stroke, management recommendations are provided to the local team. Once hemorrhage and contraindications to IV thrombolysis are excluded, thrombolysis decisions are made by the tele-stroke neurologist. Subsequent vascular imaging is guided by clinical severity at the discretion of the tele-stroke neurologist: patients with minor, non-disabling symptoms may undergo Doppler ultrasound, whereas those with disabling deficits typically receive CT angiography (CTA). Advanced perfusion imaging is not available in tele-stroke centers.

Transfer to the CSC is considered in a range of scenarios, including but not limited to confirmed indication for EVT, suspected LVO with clinico-radiological mismatch requiring advanced imaging, or risk of neurological deterioration. For patients identified as EVT candidates prior to transfer, the CSC workflow prioritizes direct-to-angio, with multimodal CT reserved for cases with significant clinical change (e.g., to exclude hemorrhage in case of deterioration or to assess recanalization in case of improvement). This protocol applies both during and outside office hours, with the neurointerventional team activated in advance and present at the time of patient arrival. In other cases, patients are reassessed by the in-house CSC neurologist, and multimodal imaging is completed as needed.

Supplemental Results

Table S1. Univariate and adjusted multivariate quantile regression for median door to EMS transfer request time

**Variable**	**median difference**	**95% CI**
Univariate		
Cohort	1.00	-7.53, 11.01
Adjusted multivariate		
Cohort	-1.15	-7.06, 3.78
Age	**0.29**	**0.15, 0.45***
Sex, male	-2.97	-7.28, 4.48
NIHSS	-1.39	-1.56, -0.61
mRS 0-2	-5.27	-33.70, 5.37
Hospital – Center A	**-19.24**	**-31.83, -12.31***
iv-Thrombolysis	-1.20	-7.17, 5.44
Arrival between 8:00 and 17:00h	2.123	-2.06, 9.07
Code activation at ED arrival (vs EMS prenotification)	**14.32**	**9.59, 27.10***
In-hospital stroke (vs EMS prenotification)	**44.09**	**19.26, 70.67***

ED, Emergency Department; EMS, Emergency Medical Services; NIHSS, National Institute of Health Stroke Scale; mRS, modified Rankin scale. *Significant values (95% CI excluding 0) are shown in bold.

Table S2. Balance of the IPTW-matched cohort

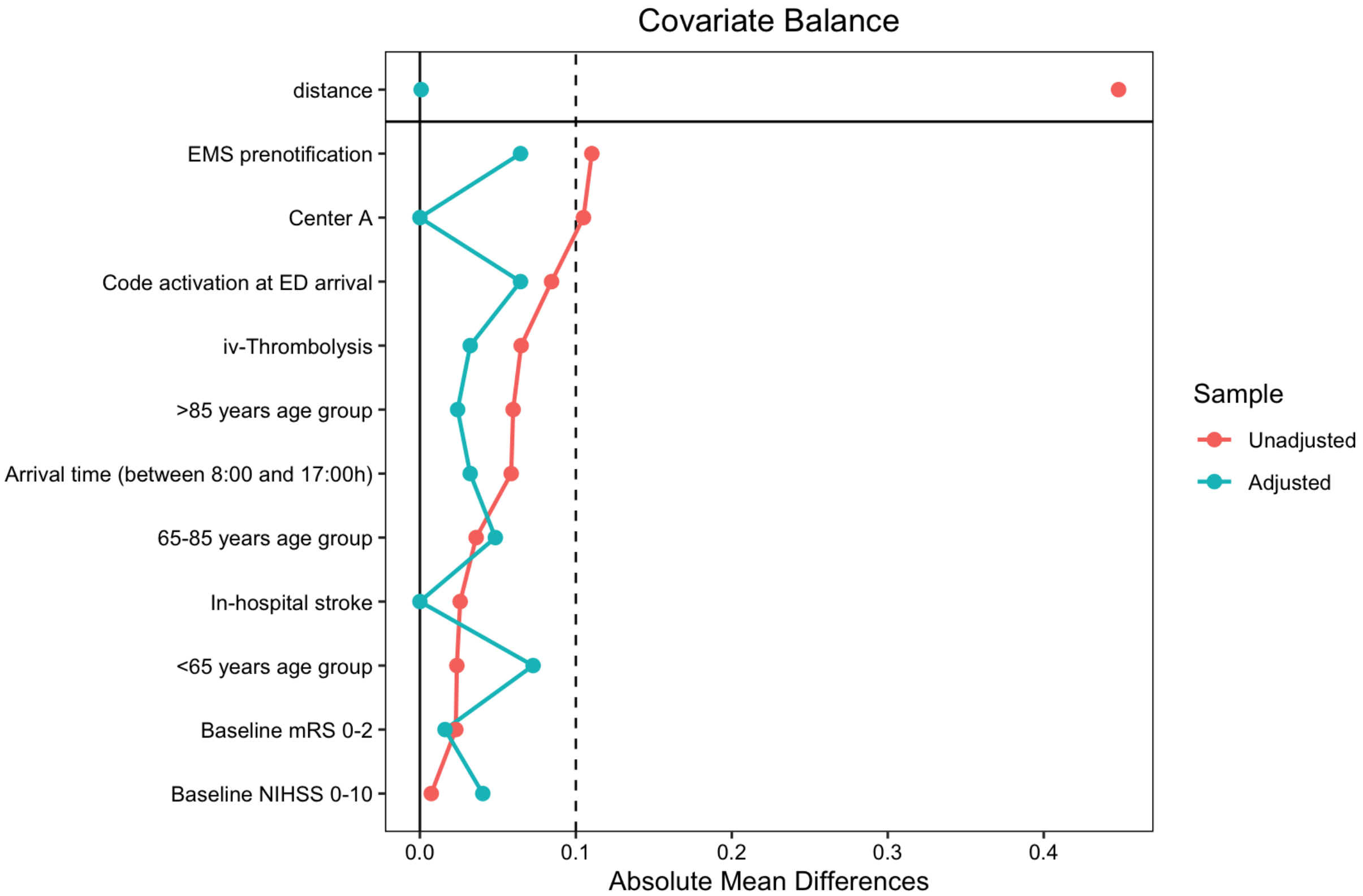

Table S2. Univariate and adjusted multivariate quantile regression for median image acquisition to EMS transfer request time

Variable	**Adjusted median difference**	**95% CI**
Cohort	**-7.16**	**-13.02, -1.85***
Age	0.13	-0-08, 0.35
Sex, male	3.36	-2.07, 10.52
NIHSS	**-0.71**	**-1.27, -0.32***
mRS 0-2	-5.62	-11.72, 4.50
Hospital – Center A	-8.92	-14.2, 2.93
iv-Thrombolysis	4.03	-4.31, 9.33
Arrival between 8:00 and 17:00h	3.35	-3.99, 8.95
Code alert at ED (vs EMS prenotification)	5.69	-5.21, 8.73
In-hospital stroke (vs EMS prenotification)	**16.94**	**0.55, 29.10***

ED, Emergency Department; EMS, Emergency Medical Services; NIHSS, National Institute of Health Stroke Scale; mRS, modified Rankin scale. *Significant values (95% CI excluding 0) are shown in bold.

Table S3. Adjusted multivariate quantile regression for median Local-SC arrival to groin puncture at the CSC

Variable	**Adjusted median difference**	**95% CI**
Cohort	**-31.28**	**-60.89, -13.74***
Age	0.33	-0.40, 1.29
Sex, male	13.30	-8.48, 48.71
NIHSS	**-3.02**	**-4.58, -0.19***
mRS 0-2	-25.4	-70.54, 11.8
Hospital – Center A	**26.91**	**1.23, 49.68***
iv-Thrombolysis	-14.92	-38.43, 6.97
Arrival between 8:00 and 17:00h	-6.40	-32.78, 20.74
Code alert at ED (vs EMS prenotification)	16.28	-17.74, 58.40
In-hospital stroke (vs EMS prenotification)	**52.11**	**7.65, 79.31***

ED, Emergency Department; EMS, Emergency Medical Services; NIHSS, National Institute of Health Stroke Scale; mRS, modified Rankin scale. *Significant values (95% CI excluding 0) are shown in bold.

Table S4. Adjusted multivariate quantile regression for median door-to-needle time in the Local-SC

Variable	**Adjusted median difference**	**95% CI**
Cohort	-0.18	-14.7, 7.58
Age	-0.07	-3.47, 0.65
Sex, male	-1.30	-1.24, 9.64
NIHSS	-1.41	-2.29, 1.49
mRS 0-2	1.46	-14.14, 1.79
Hospital – Center A	-0.97	-25.96, 9.57
Arrival between 8:00 and 17:00h	2.68	-6.57,12.81
Code alert at ED (vs EMS prenotification)	3.69	-10.60, 21.35
In-hospital stroke (vs EMS prenotification)	24.16	Inf-inf

ED, Emergency Department; EMS, Emergency Medical Services; NIHSS, National Institute of Health Stroke Scale; mRS, modified Rankin scale. *Significant values (95% CI excluding 0) are shown in bold.

Table S5. Adjusted multivariate logistic regression for CTA acquisition at the local stroke center

Variable	**aOR**	**95% CI**
Cohort	**31.16**	**13.30, 84.47***
Age	1.02	0.99, 1.05
Sex, male	0.96	0.44, 2.09
NIHSS	0.99	0.94, 2.09
mRS 0-2	1.06	0.33, 3.31
Hospital – Center A	**0.02**	**0.004-0.05***
iv-Thrombolysis	1.90	0.85-4.47

NIHSS, National Institute of Health Stroke Scale; mRS, modified Rankin scale. *Significant values (95% CI excluding 1) are shown in bold.

Table S6. Adjusted multivariate logistic regression for repeated imaging acquisition at the CSC

Variable	aOR	95% CI
Cohort	**0.47**	**0.22,0.97***
Age	0.99	0.96, 1.01
Sex, male	1.36	0.79, 2.66
NIHSS	0.99	0.94, 1.05
mRS 0-2	2.06	0.76, 5.78
Hospital – Center A	0.85	0.39, 1.81
CTA at the Local-SC	**0.13**	**0.05-0.30***
iv-Thrombolysis	0.89	0.47-1.71
Endovascular Treatment	**0.20**	**0.08-0.43***

NIHSS, National Institute of Health Stroke Scale; mRS, modified Rankin scale. *Significant values (95% CI excluding 1) are shown in bold.

Table S7. Adjusted multivariate ordinal regression for mRS at 90 days

Variable	**aOR**	**95% CI**
Cohort	1.01	0.59, 1.72
Age	**1.03**	**1.01, 1.06***
Sex, male	1.28	0.75, 2.20
NIHSS	**1.14**	**1.09, 1.19***
mRS 0-2	**0.15**	**0.06-0.34***
iv-Thrombolysis	0.63	0.38, 1.06

NIHSS, National Institute of Health Stroke Scale; mRS, modified Rankin scale. *Significant values (95% CI excluding 1) are shown in bold.

Figure S2. Detailed LVO predictions from CATalyze cohort and screening log

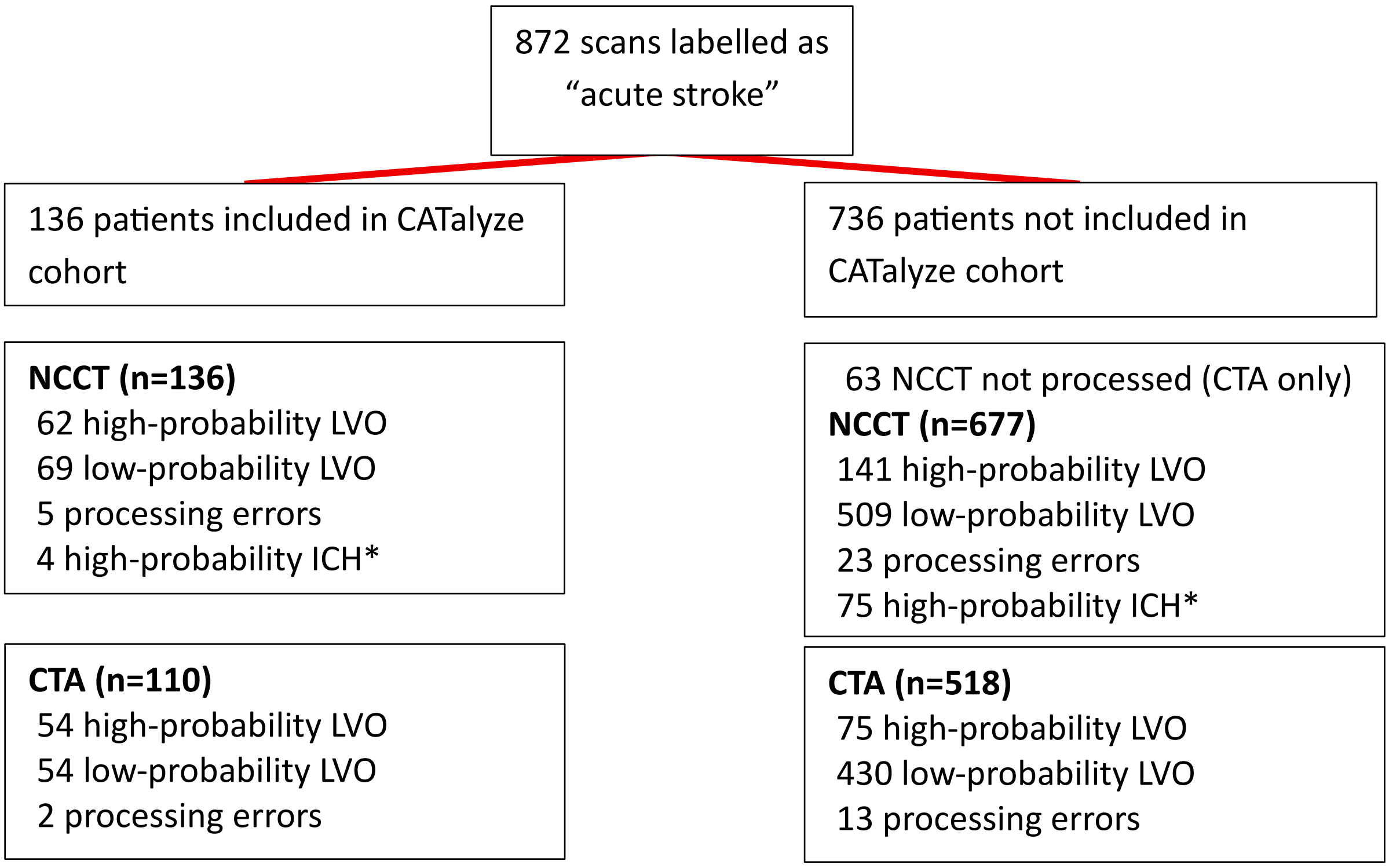

* Probability of ICH is established independently of LVO, therefore both predictions can coexist. None of the cases in the CATalyze-AI cohort and, at least, 6 cases in the non-transferred cohort labelled as high-probability ICH corresponded to a hemorrhagic stroke.

Table S8. Positive Predictive Value (PPV) of AI LVO predictions

Only patients undergoing NCCT and CTA (n=455)

	Labelled as high-probability LVO (n)	LVO* (n)	PPV	anterior LVO** (n)	PPV
NCCT					
CATalyze	62	43	69%	37	60%
Non-transferred	99	2+15†	17%††	NA	NA
Combined cohorts	161	60	37%	NA	NA
CTA					
CATalyze	54	40	74%	36	67%
Non-transferred	63	3+32‡	56%‡‡	NA	NA
Combined cohorts	117	75	64%	NA	NA

* LVO including anterior (intracranial internal carotid artery, M1 and proximal M2 segments of middle cerebral artery) and posterior (intracranial vertebral arteries, basilar artery and P1 segment of posterior cerebral artery). It should be noted that the algorithm does not target posterior circulation occlusions. ** In the screening log, LVO location is not identified.

† In the non-transferred NCCT cohort, 3 patients had a confirmed LVO, LVO was excluded in 82 cases according to the screening log, and in the remaining 15 cases, definitive diagnosis is unknown. †† For PPV estimation, a best-case scenario was assumed in which all patients with unknown status were considered true LVOs (2+15)

‡ In the non-transferred cohort, 3 patients had a confirmed LVO, LVO was reasonably excluded in 28 patients according to the screening log, and in the remaining 32 cases, definitive diagnosis is unknown. ‡‡ For PPV estimation, a best-case scenario was assumed in which all patients with unknown status were considered true LVOs (3+32)
